# Short-Term and Long-Term Renal Outcomes in Patients With Obesity After Minimally Invasive Versus Open Partial Nephrectomy for the Treatment of Renal Cancer: Retrospective Study

**DOI:** 10.2196/19750

**Published:** 2022-01-10

**Authors:** Brittany Flippo, Bradley Stone, Shelbie Stahr, Mahmoud Khalil, Rodney Davis, Mohamed Kamel, Manisha Singh

**Affiliations:** 1 University of Arkansas for Medical Sciences Little Rock, AR United States

**Keywords:** renal outcomes, renal cell carcinoma, minimally invasive vs open partial nephrectomy, obesity, kidney, cancer, surgery, retrospective, outcome, short-term, long-term

## Abstract

**Background:**

Obesity is significantly associated with renal cell carcinoma. Surgery is the preferred treatment for demarcated lesions of renal cell carcinoma; however, obesity increases the complexity of surgical outcomes. Minimally invasive surgical techniques are preferred over open partial nephrectomy (OPN), but controversy remains regarding the most efficacious technique in patients with obesity.

**Objective:**

This study aims to determine whether minimally invasive partial nephrectomy (MIPN) or OPN better preserves renal function and investigate short- and long-term renal outcomes in patients with obesity undergoing a partial nephrectomy.

**Methods:**

We conducted a retrospective chart review of 242 adult patients aged ≥18 years who underwent MIPN or OPN between January 1, 2005, and December 31, 2016, at the University of Arkansas for Medical Sciences. Using creatinine as a measure of kidney function, patients’ preoperative levels were compared with their postoperative levels in 2-time frames: short (3-6 months postsurgery) or long (>6 months). The primary outcome was the change in creatinine values from preoperative to >6 months postoperatively in patients with obesity. Secondary outcomes included the change in creatinine values from preoperative to 3 to 6 months postoperatively in patients with obesity who underwent MIPN versus OPN. We also analyzed the creatinine values of nonobese patients (BMI <30) who underwent partial nephrectomy using the same time frames. Unconditional logistic regression was used to estimate crude and multivariable-adjusted odds ratios (ORs) and 95% CI to observe associations between surgery type and changes in creatinine values from while stratifying for obesity.

**Results:**

A total of 140 patients were included in the study, of whom 75 were obese and 65 were nonobese. At >6 months after MIPN (n=20), the odds of patients with obesity having a decrease or no change in creatinine values was 1.24 times higher than those who had OPN (n=13; OR 1.24, 95% CI 0.299-6.729; *P*=.80). At 3 to 6 months after MIPN (n=27), the odds were 0.62 times lower than those after OPN (n=17; OR 0.62, 95% CI 0.140-2.753; *P*=.56). In the nonobese group, at 3 to 6 months after undergoing minimally invasive surgery (n=18), the odds of having a decrease or no change in creatinine values was 4.86 times higher than those who had open surgery (n=21; OR 4.86, 95% CI 1.085-21.809; *P*=.04). At more than 6 months after MIPN (n=14), the odds were 4.13 times higher than those after OPN (n=11; OR 4.13, 95% CI 0.579-29.485; *P*=.16).

**Conclusions:**

We observed a nonstatistically significant preservation of renal function in patients with obesity who underwent OPN at 3 to 6 months postoperatively. Conversely, after 6 months, the same was true for MIPN, indicating the long-term benefit of MIPN. In the nonobese group, MIPN was favored over OPN.

## Introduction

### Background

Because of the negative impact obesity has on quality of life coupled with the associated increase in morbidity and mortality, obesity is one of the most significant modifiable health issues facing the United States and is reaching epidemic proportions [1-5]. As of 2016, obesity affects 13% adults worldwide, of which 39.8% are in the United States alone [6,7]. Furthermore, obesity is a major risk factor for other comorbidities, such as diabetes, heart disease, and cancer [8-12]. Particularly, the chance of acquiring renal cell carcinoma (RCC) is significantly higher in the obese population [13-15]. RCC is one of the top 10 cancers diagnosed in the United States. It is estimated that there will be 76,080 new cases of kidney cancer in 2021, which is higher than the estimated value for 2020 [16].

To preserve nephrons and, in turn, preserve renal function, partial nephrectomy has become the preferred option to radical nephrectomy when removing small renal tumors [14,17-19]. Although the more traditional open partial nephrectomy (OPN) is still performed, many have opted for minimally invasive surgical techniques, such as laparoscopic partial nephrectomy (LPN) or robotic partial nephrectomy (RPN), because of their shorter hospital stays and operating times [20]. With increased precision, dexterity, and 3D capabilities, RPNs are more commonly performed than OPNs or LPNs [21,22].

As obesity rates continue to rise, the treatment and management of renal cancer in the obese population poses a unique challenge. Because of the increased likelihood of postoperative complications, both medical and surgical techniques must be assessed to establish the best practice for patients with obesity. Although increased BMI adds another level of difficulty for physicians performing partial nephrectomies, the procedure is considered especially advantageous in this population [20,23]. RPNs have become a more favorable approach in the general population; however, there is still controversy regarding the most efficacious surgical technique in patients with obesity. Furthermore, several studies have found comparable short-term (<3 months postoperatively) outcomes in kidney function between patients with without obesity who underwent partial nephrectomy, but there are very limited data on long-term kidney function (>6 months postoperatively) [24,25].

### Objective

Because patients with obesity are already at risk of chronic kidney disease (CKD), maintaining renal function is a high priority [26]. Our goal is to investigate long-term renal outcomes in patients with obesity for up to 4 years after undergoing either MIPN or OPN. The gold standard for measuring renal function and diagnosing CKD is the estimated glomerular filtration rate (eGFR). However, there is still evidence that eGFR using the CKD epidemiology collaboration equation is not accurate in patients with obesity with a BMI >40 [27]. Because patients with obesity are our target study population, we decided to compare patients’ baseline creatinine levels before surgery to creatinine levels at 3 to 6 months (short term) and >6 months (long term) postoperatively. Although creatinine should not be used as the only factor to determine kidney health, our main goal is to determine whether patients maintained or improved their kidney function. Serum creatinine can be affected by age, race, sex, and BMI; therefore, we controlled and adjusted for these variables in our statistical model. On the basis of our results, we hope to contribute data regarding the optimal surgical approach, offering improved preservation of long-term kidney function in the obese population. This information may provide guidance to surgeons when deciding which surgical approach is the most appropriate for this population.

## Methods

### Study Design

The Institutional Review Board of University of Arkansas for Medical Sciences (UAMS) approved a retrospective chart review, conducted to identify patients with obesity aged ≥18 years who underwent OPN, LPN, or RPN for the treatment of renal cancer between January 1, 2005, and December 31, 2016, at UAMS. This study was conducted in accordance with all applicable government regulations and UAMS research policies and procedures. This retrospective study used existing data, and there was no direct contact with the study participants. A waiver of informed consent was approved by the institutional review board as there was no more than minimal risk to the participants, and the waiver did not adversely affect the rights or welfare of the subjects. The longest follow-up data that we found in patient charts were for 4 years, and the number of patients who had data this far out postoperatively was limited.

### Patient Selection 

Originally used in the study by Webb et al [25], a previously existing institutional data set of partial nephrectomies was updated using patient charts from January 1, 2005, to May 4, 2011. A new subset of patients from May 5, 2011, to December 31, 2016, was added to the existing institutional database. All patient data, including both demographic and surgical data, were collected by retrospective chart review using an electronic medical records system. Patients were deidentified, and data were stored in a passcode-protected Microsoft Excel file. A system set was in place to find the specific information needed for each patient, so all data entries were consistent. The study was conducted in 2018. As this was an existing database and we wanted to analyze these data with a longer timeline (>6 months), we included patients who underwent surgery until 2016 to ensure that enough time was allotted postoperatively to evaluate long-term data. If patients who underwent surgery in 2017 or 2018 were included, many may not have fallen into the correct timeline. Patients were excluded if they had a previous radical nephrectomy, or if a planned partial nephrectomy was converted to a radical nephrectomy intraoperatively. A total of 242 patients were identified who met the criteria of being >18 years and having undergone a partial nephrectomy for the treatment of renal cancer. Patients were further excluded if they did not have a preoperative creatinine value, a postoperative creatinine value, or if a full medical history could not be found, which would list any comorbidities.

### Surgical Approach

During this time, 3 urologists performed partial nephrectomies. All patients were consulted by a urologist in the clinic to determine whether OPN, LPN, or RPN (with LPN and RPN combined together as minimally invasive or MIPN) would be the most effective surgical approach for the patient. All 3 surgeons performed all the 3 surgical approaches. Decisions were made as per patient consent from discussions with their urologist regarding the risks versus benefits of each approach. The minimally invasive technique of choice was purely laparoscopic from 2005 to 2007. There were no laparoscopic surgeries after 2014, as there was a shift from the laparoscopic approach to the robotic approach. The shift began in 2008, with gradually more robotic surgeries and fewer laparoscopic surgeries. Both approaches are considered minimally invasive. With our timeline spanning the era of laparoscopic surgery, the slow transition to robotic surgery, and the era of almost exclusively robotic surgery as the minimally invasive approach, it was necessary to combine the cohorts. As with any new surgical technique, a learning curve needs to be considered. All patients were scheduled for regular outpatient follow-up after partial nephrectomies, regardless of the approach. No group was scheduled to be followed more or less frequently. Most follow-ups included an iStat creatinine blood draw or a basic metabolic panel.

### Primary Outcome

The primary outcome was the change in creatinine values from preoperative to >6 months postoperatively in patients with obesity who underwent MIPN versus those who underwent OPN. Preoperative creatinine values were obtained for every patient, and subsequent creatinine values were followed up for 4 years.

### Secondary Outcomes

Secondary outcomes included the change in creatinine values from preoperative to 3-6 months postoperatively in patients with obesity who underwent MIPN versus those who underwent OPN. These findings were used to analyze the short-term effects on renal function. The change in creatinine values from preoperative to 3-6 months and >6 months postoperatively in patients without obesity who underwent MIPN or OPN were also studied as a secondary outcome. In both patient populations, differences in patient characteristics, tumor location, tumor size, pathology, age, average clamp time (defined as the time from which the renal artery was clamped to the time it was unclamped), estimated blood loss, and length of hospital stay were analyzed.

### Statistical Analysis 

Various analyses were performed on the patients’ creatinine concentrations. Creatinine values preoperatively, at discharge, 3 to 6 months postoperatively, and at >6 months after discharge were dichotomized into either <1 mg/dL or ≥1 mg/dL. Dummy variables were created for creatinine values during the 3- to 6-month and >6-month postoperative periods by subtracting the postoperative creatinine levels from the preoperative creatinine levels. A second dummy variable was created to record the difference in creatinine from preoperative to its corresponding postoperative time. The dichotomous variable indicated whether the individual’s creatinine level had increased, decreased, or had no change since the preoperative period.

Differences in patient characteristics between surgical approach types were evaluated using chi-square tests for categorical variables and *t* tests for continuous variables (Table 1). BMI was calculated using the following formula: BMI=(weight [kg]/height [m^2^]). BMI of <30 was defined as nonobese and BMI of ≥30, as obese. Variables evaluating sex (male and female) and race (European American and African American) were treated as dichotomous variables, and those evaluating age, as a continuous variable. Comorbidities hypertension, diabetes mellitus, prior kidney complications, and smoking status were combined to create 1 ordinal comorbidity variable. Each of the abovementioned comorbidities was labeled as a yes-no variable, except for smoking, which had the addition of a *prior smoking* label. These variables were then summed according to the total number of yes responses (and the number of prior responses for smoking) to create a single, continuous comorbidity variable and limit the multicollinearity of these comorbidities.

**Table 1:** Patient characteristics by surgical approach and obesity status.

Characteristic		Obese				Nonobese		
		MIPN^a^ (n=50)	OPN^b^ (n=25)	*P* value	MIPN (n=36)		OPN (n=29)	*P* value
**Age (years), mean (SD)**		57 (10.63)	55 (11.67)	.38	56 (14.73)		58 (12.96)	.51
	Missing, n^c^	0^d^	0		0		0	
**Race**				.65				.20
	European American, n (%)	43 (87.76)	21 (84.00)		27 (84.38)		26 (96.30)	
	African American, n (%)	6 (12.24)	4 (16.00)		5 (15.62)		1 (3.70)	
	Missing, n^c^	1	0		4		2	
**Sex**				.41				.22
	Male, n (%)	27 (54.00)	16 (64.00)		24 (66.67)		15 (51.72)	
	Female, n (%)	23 (46.00)	9 (36.00)		12 (33.33)		14 (48.28)	
	Missing, n^c^	0	0		0		0	
**Tumor location**				.045				.85
	Left, n (%)	26 (52.00)	19 (76.00)		17 (47.22)		13 (44.83)	
	Right, n (%)	24 (48.00)	6 (24.00)		19 (52.78)		16 (55.17)	
	Missing, n^c^	0	0		0		0	
**Tumor size (cm), mean (SD)**		2.85 (1.65)	3.97 (2.15)	.04	3.25 (1.76)		3.58 (1.54)	.41
	Missing, n^c^	2	2		0		1	
**Pathology**				.18				.26
	Benign, n (%)	10 (20.00)	2 (8.00)		9 (25.00)		4 (13.79)	
	Malignant, n (%)	40 (80.00)	23 (92.00)		27 (75.00)		25 (86.21)	
	Missing, n^c^	0	0		0		0	
**Stage**				.37				.83
	Stage 1, n (%)	33 (66.00)	21 (84.00)		23 (63.88)		18 (62.06)	
	Stage 2, n (%)	3 (6.00)	1 (4.00)		2 (5.56)		2(6.90)	
	Stage 3, n (%)	2 (4.00)	1 (4.00)		0 (0.00)		1 (3.45)	
	Stage 4, n (%)	0 (0.00)	0 (0.00)		0 (0.00)		1 (3.45)	
	Missing, n^c^	12	2		9		7	
**Length of stay (days), mean (SD)**		3 (1.31)	5 (3.33)	<.001	3 (1.81)		4 (1.29)	.08
	Missing, n^c^	0	0		0		0	
**Average clamping time (min), mean (SD)**		24.00 (11.79)^e^	31.36 (11.47)^f^	.05			31.43 (11.08)^e^	.007
	Missing, n^c^	11	11		4		8	
**Estimated blood loss (dL), mean (SD)**		132.50 (238.74)	325.00 (338.63)	<.001			275.00 (271.69)	.07
	Missing, n^c^	0	1		0		2	

^a^MIPN: marginally invasive partial nephrectomy.

^b^OPN: open partial nephrectomy.

^c^Number of patients without a recorded variable.

^d^Missing data has been entered as 0.

^e^Warm ischemia.

^f^Cold ischemia.

Unconditional logistic regression was used to estimate crude and multivariable-adjusted odds ratios (ORs) and 95% CIs to observe associations between surgery type and changes in creatinine values from preoperative concentrations while stratifying for obesity. Logistic regression was used because of the small sample size limitations, amplified by the stratification of obesity. Creatinine values were not normally distributed; therefore, logistic regression was used to observe the relationship between creatinine differences and the 2 surgery types. Potential confounding variables for the association between surgery type and changes in creatinine level were included in the multivariable logistic regression models. Confounding variables were selected based on prior knowledge and a 10% change in the beta coefficient (results not shown). Although covariates such as length of stay in the hospital, average clamping time, and estimated blood loss were statistically different (Table 1), they could not be included in the model because of the model’s inability to converge due to small sample sizes. The most parsimonious model was used to estimate associations between surgery type and changes in creatinine while stratifying by obesity status, with covariates including age, sex, race, comorbidities, and preoperative creatinine values. Each analysis was 2-sided, with *P* values of less than .05. All analyses were performed using the SAS version 9.3 (SAS Institute).

### Sensitivity Analysis

A sensitivity analysis was performed to determine whether LPN and RPN produced similar results; however, the models could not converge because of the small sample size. If the patients who underwent LPN were removed, then the sample size would not be large enough to run the analysis.

## Results

### Overview

After controlling for race, sex, age, comorbidities, and available creatinine values, data for 140 patients, of whom 75 were obese and 65 were nonobese, were analyzed.

As presented in Table 1, there were statistically significant differences in tumor location (*P*=.045), tumor size (*P*=.04), length of stay (*P≤*.001), and estimated blood loss (*P≤*.001) in patients with obesity who underwent MIPN versus those who underwent OPN. Patients without obesity who underwent MIPN demonstrated a statistically significant (*P=*.007) shorter average clamp time than patients without obesity who underwent OPN. Patients who underwent MIPN had warm ischemia, whereas patients who underwent OPN had cold ischemia during tumor resection.

Table 2 shows mean creatinine values of obese and patients without obesity preoperatively, at discharge, 3 to 6 months postoperatively, and at >6 months after OPN or MIPN. As noted in Table 3, 44 patients with obesity and 39 patients without obesity had creatinine levels at 3 to 6 months. Furthermore, Table 4 shows that 33 patients with obesity and 25 patients without obesity had creatinine levels at >6 months.

**Table 2:** Patient mean creatinine values by surgical approach and obesity status.

Period				Obese						Nonobese				
				MIPN^a^ (n=50)		OPN^b^ (n=25)		*P* value		MIPN (n=36)		OPN (n=29)		*P* value
**Preoperative creatinine (mg/dL), mean (SD)**				0.96 (0.36)		1.12 (0.50)		.11		0.97 (0.35)		1.06 (0.42)		.38
	Missing, n^c^		0^d^		0				0		0			
**Creatinine at discharge (mg/dL), mean (SD)**				1.11 (0.45)		1.45 (1.38)		.11		1.12 (0.41)		1.01 (0.39)		.30
	Missing, n^c^			1		0				0		1		
**Creatinine at 3-6 months (mg/dL), mean (SD)**				1.10 (0.292)		1.41 (0.858)		.17		1.06 (0.483)		1.03 (0.408)		.83
	Missing, n^c^			23		8				18		8		
**Creatinine at >6 months (mg/dL), mean (SD)**				1.12 (0.303)		1.16 (0.661)		.81		1.14 (0.648)		0.95 (0.333)		.38
	Missing, n^c^			30		12				22		18		

^a^MIPN: minimally invasive partial nephrectomy.

^b^OPN: open partial nephrectomy.

^c^Number of patients without a recorded variable.

^d^Missing data have been entered as 0.

**Table 3:** Change in 3- to 6-month creatinine values.

Obesity status	Unadjusted					Adjusted^a^			
	Participants, n (%)	Surgery type	OR^b^ (95% CI)	*P* value	Participants, n (%)		Surgery type	OR (95% CI)	*P* value
**Obese**									
	17 (68)	OPN^c^	1 (reference)	N/A^d^	17 (68)		OPN	1 (reference)	N/A
	27 (54)	MIPN^f^	0.83 (0.224-3.103)	.79	27 (54)		MIPN	0.62 (0.140- 2.753)	.56
**Nonobese**									
	21 (72)	OPN	1 (reference)	N/A	20 (69)		OPN	1 (reference)	N/A
	18 (50)	MIPN	5.20 (1.317-20.539)	.02	18 (50)		MIPN	4.86 (1.085-21.809)	.04

^a^Adjusted for age, sex, race, comorbidities, and preoperative creatinine values.

^b^OR: odds ratio.

^c^OPN: open partial nephrectomy.

^d^N/A: not applicable.

^e^MIPN: minimally invasive partial nephrectomy.

**Table 4:** Change in <6 month creatinine values.

Obesity status	Unadjusted					Adjusted^a^			
	Participants, n (%)	Surgery type	OR^b^ (95% CI)	*P* value	Participants, n (%)		Surgery type	OR (95% CI)	*P* value
**Obese**									
	13 (52)	OPN^c^	1 (reference))	—	13 (52)		OPN	1 (reference)	—
	20 (40)	MIPN^e^	2.17 (0.521-9.017)	.29	20 (40)		MIPN	1.24 (0.229-6.729)	.80
**Nonobese**									
	11 (38)	OPN	1 (reference)	—	11 (38)		OPN	1 (reference)	—
	14 (39)	MIPN	3.56 (0.651-19.412)	.14	14 (39)		MIPN	4.13 (0.579-29.485)	.16

^a^Adjusted for age, sex, race, comorbidities, and preoperative creatinine values.

^b^OR: odds ratio.

^c^OPN: open partial nephrectomy.

^d^N/A: not applicable.

^e^MIPN: minimally invasive partial nephrectomy.

### Primary Analysis

For our primary outcome, at >6 months after minimally invasive surgery (n=20), the odds of patients with obesity having a decrease or no change in creatinine values were 1.24 times higher than those who had open surgery (n=13). However, this difference was not statistically significant (*P*=.80).

### Secondary Analysis

At 3 to 6 months after minimally invasive surgery (n=27), the odds of patients with obesity having a decrease or no change in creatinine values were 0.62 times lower than those who had open surgery (n=17); however, this too was not statistically significant (*P*=.56).

At 3 to 6 months after minimally invasive surgery (n=18), the odds of patients without obesity having a decrease or no change in creatinine values were 4.86 times higher than those who had open surgery (n=21), which was statistically significant (*P*=.04). At >6 months after minimally invasive surgery (n=14), the odds of patients without obesity having a decrease or no change in their creatinine values were 4.13 times higher than those who had open surgery (n=11), which was not statistically significant (*P*=.16).

## Discussion

### Principal Findings

When evaluating the efficacy of OPN and MIPN using creatinine levels postoperatively, our results did not show a significant difference in long-term renal function in patients with obesity. However, our results showed a nonsignificant improvement in renal function at 3 to 6 months postoperatively in patients with obesity who underwent OPN. Conversely, after 6 months postoperatively, there was a nonsignificant improvement in renal function in patients with obesity who underwent MIPN. Although our study set out to observe patients with obesity after these different surgical approaches, we also observed patients without obesity as a secondary outcome and found a statistically significant result regarding neutral or better renal function after MIPN. In the nonobese group, MIPN at 3 to 6 month and >6 months postoperatively was favored over OPN, with only the 3- to 6-month range being statistically significant.

We hypothesize that patients without obesity are able to compensate better and earlier with the nonsurgical kidney because they are more likely to be healthier and have fewer comorbidities. In addition, the patients without obesity who underwent MIPN had a more significantly reduced clamp time than that of patients without obesity who underwent OPN, which could have contributed to better renal function. Other possible explanations for our results include the effects of pneumoperitoneum on renal function, impact of the type of ischemia, learning curve in performing MIPN in the early study period, and the small sample size. When selecting follow-up periods for establishing what defines as *long-term*, the urology department was consulted; it was agreed that 6 months was a good break point for long-term kidney function because short term is most often determined as 2 to 3 months post operation. Because there is no formal structure for long-term follow-up after a partial nephrectomy, it is possible that the experience that the surgeons had with patients losing follow-up between 6 months and 1 year could have contributed to the time periods they recommended for analysis. When choosing the cutoff for BMI, we recognized that there could be differences between obesity and severe obesity; however, we did not have the sample size to break down the data into further groups. It would be ideal for future studies to have a larger sample size to assess more specific obesity categories.

Although partial nephrectomy has become the preferred option over radical nephrectomy for small renal tumors, a reduction of approximately 20% in renal function has been found in patients undergoing partial nephrectomies [14,17-19,28,29]. The amount of remaining healthy kidney tissue after nephrectomy is recognized as the most important factor for future renal function [30]. There have been conflicting results in recent literature comparing long-term renal function after MPIN with OPN. When comparing 866 patients undergoing either OPN or RPN, Yu et al [31] found that RPN preserved renal function better by analyzing preoperative eGFR to postoperative eGFR at 6 to 8 months. Another study by Wang et al [32] compared 360 patients undergoing either OPN or RPN showed no difference in eGFR over the long term. However, they specifically examined complex renal tumors. Furthermore, Choi et al [33] found that eGFR using diethylenetriamine pentaacetate renal scintigraphy was lower after open surgery than after robotic surgery when analyzing data up to 1 year postoperatively. However, 1 to 4 years postoperatively, eGFR between the 2 groups was comparable. When specifically examining long-term renal function in patients with obesity after partial nephrectomy, existing literature is limited and conflicting. One multi-institutional retrospective review evaluated long-term renal function and CKD predictors 1 year after MIPN in patients with obesity and did not find BMI or operative technique as a predictor of progression to CKD [20]. Another study comparing the outcomes of 237 patients with obesity undergoing either RPN or OPN found no significant difference in eGFR between the 2 surgical groups. The median eGFR follow-up time was approximately 2 years, indicating some insight into long-term renal function [34]. Several studies have evaluated short-term complications in the obese population, but data on long-term kidney function are lacking [24,25]. One of the reasons we believe the results are so scarce and inconsistent is that there is no set protocol standard in the assessment of successful outcomes of surgery. Some studies focused on intraoperative complications, while others looked at postoperative complications, and others used readmission as their only qualification. 

### Limitations

Our study is retrospective, which is a limitation of our study. No group was scheduled for follow-up in clinic more or less than another; however, many patients stopped showing up for their appointments, and the reason for this is unknown because this study was retrospective. Another limitation of retrospective studies is the risk of bias. We aimed to address selection bias, measurement bias, and confounding bias to decrease the effect of these biases during a retrospective study. To address selection bias, every patient aged >18 years who underwent a partial nephrectomy for the treatment of renal cancer were eligible for inclusion. The decision for partial nephrectomy versus radical nephrectomy could not be controlled because this was a retrospective study; however, the most desirable approach at this institution was partial nephrectomy if possible. The surgical approach of each partial nephrectomy (MIPN vs. OPN) could not be controlled or randomized, given the nature of the study. The decision for the approach was made between the patient and the physician.

To address measurement bias, every patient included in the study analysis had a preoperative creatinine level and postoperative creatinine level. Patients were assessed in groups depending on the time frame of the postoperative creatinine values available in their charts. We divided patients in 2 groups by 3- to 6-month postoperative, which is considered a short-term outcome in renal function and >6-month postoperative, which is considered long term. Preoperative and postoperative creatinine values were checked multiple times to ensure that the correct values were entered into the database. If these values were incorrect, the results would be affected because the main outcome depended on calculating the difference in preoperative and postoperative creatinine values.

To address confounding bias, we controlled for comorbidities, especially those that affect kidney function, that are common among the obese population. Just as there is an increased risk of RCC in the obese population, there is also a higher risk for diabetes and hypertension, both of which can affect kidney function. When gathering data, we documented pre-existing health conditions for each patient, including diabetes and hypertension, and smoking status. These comorbidities were controlled for during statistical analysis. If the above confounding variables were not controlled for, we could not be certain that the changes in creatinine were due to the surgical approach. Patients were excluded if they had a previous radical nephrectomy, as this would dramatically affect the results; however, we did not control for other previous abdominal surgeries, which may have contributed to some bias.

In addition, 3 different surgeons performed all 3 surgical approaches (open, laparoscopic, and robotic) to partial nephrectomy at our institution during this period. All 3 surgeons were trained at different locations. Surgeons’ techniques and experiences may have contributed to some bias. Considering the importance and impact of these procedures, a prospective study is needed. This would help attrition, and it would also help us track the loss of attrition. With a prospective study, we could better follow up recurrence rates, creatinine values, eGFR, proteinuria, and newly acquired medical conditions.

Another limitation of our study was that we were unable to assess the amount of lost renal volume during surgery, which is an important predictor of long-term renal function. For a surgeon, the primary focus is to preserve renal function while optimally decreasing the tumor burden. It would be helpful to know exactly how much renal volume was lost in each case, which would only be available consistently if we had conducted a prospective study.

### Future Work

For academic purposes, we would like to observe eGFR and creatinine levels in every patient during follow-up after a partial nephrectomy for at least 1 year and extend this period for as long as possible. Anticipated barriers are the cost of time, overuse of resources, and attrition. The lack of an integrated system and follow-up protocols make it difficult to follow these patients long-term. With the understanding that there will be some limitations as there are multiple individual variables, we hope for a prospective study and a meta-analysis to help determine a surgical approach that is superior in preserving the greatest amount of long-term renal function in patients with obesity. It is important to examine both short-term and long-term outcomes to reveal a more optimal surgical approach that would decrease the risk of CKD in this susceptible population. 

### Conclusion

When evaluating the efficacy of OPN and MIPN using creatinine levels postoperatively, our results did not show a significant difference in long-term renal function in patients with obesity.

## Abbreviations

CKD: chronic kidney disease
eGFR: estimated glomerular filtration rate
LPN: laparoscopic partial nephrectomy
MIPN: minimally invasive partial nephrectomy
OPN: open partial nephrectomy
OR: odds ratio
RCC: renal cell carcinoma
RPN: robotic partial nephrectomy
UAMS: University of Arkansas for Medical Sciences
